# Speedup of quantum evolution of multiqubit entanglement states

**DOI:** 10.1038/srep27349

**Published:** 2016-06-10

**Authors:** Ying-Jie Zhang, Wei Han, Yun-Jie Xia, Jian-Xiang Tian, Heng Fan

**Affiliations:** 1 Shandong Provincial Key Laboratory of Laser Polarization and Information Technology, Department of Physics, Qufu Normal University, Qufu 273165, China; 2 Beijing National Laboratory of Condensed Matter Physics, Institute of Physics, Chinese Academy of Sciences, Beijing 100190, China; 3 Innovative Center of Quantum Matter, Beijing 100190, China

## Abstract

As is well known, quantum speed limit time (QSLT) can be used to characterize the maximal speed of evolution of quantum systems. We mainly investigate the QSLT of generalized N-qubit GHZ-type states and W-type states in the amplitude-damping channels. It is shown that, in the case *N* qubits coupled with independent noise channels, the QSLT of the entangled GHZ-type state is closely related to the number of qubits in the small-scale system. And the larger entanglement of GHZ-type states can lead to the shorter QSLT of the evolution process. However, the QSLT of the W-type states are independent of the number of qubits and the initial entanglement. Furthermore, by considering only *M* qubits among the N-qubit system respectively interacting with their own noise channels, QSLTs for these two types states are shorter than in the case *N* qubits coupled with independent noise channels. We therefore reach the interesting result that the potential speedup of quantum evolution of a given N-qubit GHZ-type state or W-type state can be realized in the case the number of the applied noise channels satisfying *M* < *N*.

Quantum mechanics establishes the fundamental and important bounds for the evolution time to transfer a given initial quantum state to a prescribed final state in a controlled and optimal way. Bounds of this evolution time, known as quantum speed limit time (QSLT), are intimately related to the maximal evolution speed of quantum systems. The utility of these limits involve in many areas of research such as the communication speed in quantum communication^1^,^2^, the precision limits in quantum metrology^3^, the computation speed in quantum computation^4^, nonequilibrium thermodynamics^5^, as well as quantum optimal control protocols^6^,^7^,^8^,^9^,^10^. Derivations of the QSLTs usually consider that such quantum systems are noiseless and undergoing unitary evolutions^11^,^12^,^13^,^14^,^15^,^16^,^17^,^18^,^19^,^20^,^21^,^22^. Since the relevant influence of the environment on processing or information transferring systems can not be ignored, recently, the bounds of evolution time including both Mandelstam-Tamm (MT) and Margolus-Levitin (ML) types focused on the open system with nonunitary dynamics process have also been formulated^23^,^24^,^25^,^26^,^27^,^28^,^29^,^30^,^31^,^32^,^33^. The QSLTs have already been used to illustrate the quantum evolution speed for a qubit state under nonunitary dynamics process. Some theoretical studies have shown that the non-Markovianity of the noise channels can speed up the quantum evolution process^26^,^29^,^30^, and this phenomenon has also been experimentally confirmed by the controlled environment^32^.

The speedup evolution of quantum state gained when using quantum system to process information should be considerable in the limit of large-scale information processing^34^,^35^,^36^, so it is significant to understand the scaling properties of the QSLT for multiqubit system. So far, a few studies have been done on the QSLT in the multiqubit systems^17^,^23^,^31^,^37^,^38^,^39^, it has been shown that entanglement could accelerate the evolution of the closed quantum system. For the multiqubit open system, in refs 23 and 24, the authors mainly consider Markovian dephasing of N-qubits system where each qubit interacts only with its own noise channel. By choosing the initial GHZ-type states, they have found that the evolution speeds of separable and entangled states scale in the same way with respect to the number of qubits, and the speedup evolution due to entanglement is also true in the nonunitary dephasing channels. But the influences of entanglement and the number of qubits on the quantum evolution speed of the multiqubit system with different types initial states under more general and typical nonunitary noise channels, are not well studied until now. So the task to explore the quantum evolution speed of the multiqubit entanglement open system is still extremely necessary.

In this paper, we investigate the QSLT of generalized N-qubit GHZ-type states and W-type states in two different cases. one case is described that *N* qubits would be coupled with independent amplitude-damping channels as shown in Fig. 1(a). In this case, the GHZ-type entanglement can reduce the QSLT of the evolution process. In addition, the QSLTs of the entangled GHZ-type states increase as the number of qubits increasing in the small-scale system, and are unaffected by the number of qubits in the larger-scale system. However, the QSLTs for the W-type states are independent of the number of qubits and the initial entanglement. The above results are obviously different from the one corresponding to the dephasing channels^23^,^24^. In the previous studies^23^,^24^,^31^, the QSLT of open multiqubit system has been analyzed in the case each qubit respectively interacting with its own noise channel. In comparison, we also consider the other case that only *M* amplitude-damping channels respectively added to *M* qubits among the N-qubit system (*M* < *N*) in Fig. 1(b). By investigating the influence of the number of the applied noise channels on the QSLT, it is striking to find that the speedup evolution of these two types states can occur. Additionally, the number of the applied noise channels *M* plays the opposite effect on the QSLTs for the mulitqubit GHZ-type states and W-type states, i.e., the shortest QSLT of a given N-qubit GHZ-type state can be acquired by choosing the case *M* = *N* − 1, while for a given N-qubit W-type state, the smallest QSLT occurs by taking the case *M* = 1.

## Results

### Decoherence model and quantum speed limit time

Here, we mainly consider *N* qubits of ground state |0〉 and excited state |1〉 without interacting with each other. *M* (1 ≤ *M* ≤ *N*) qubits respectively couple to their own amplitude-damping channels, while the other *N* − *M* qubits are isolated from any environment and would not evolve at all in the dynamical process. The dynamics of the whole system can be obtained simply from the evolution of the individual qubit. And the dynamics of the *i*-th qubit, 1 ≤ *i* ≤ *M*, can be governed by a master equation that gives rise to a completely positive trace-preserving channel Λ_*i*_ describing the evolution as 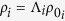, where 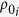 and *ρ*_*i*_ are the initial and evolved reduced states of the *i*-th qubit, respectively. In the Born-Markov approximation, the amplitude-damping channel is given by its Kraus representation as^40^ 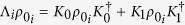, with 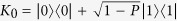 and 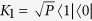. In the zero-temperature limit, *P* = *e*^−Γ*t*^ is the decay of the excited population, and Γ is the dissipation rate.

Let us consider the situation where the initial state of *N* qubits is in the multiqubit GHZ-type state or W-type state, that is 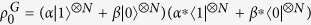, or 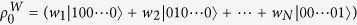

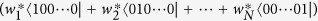
, with |*α*|^2^ + |*β*|^2^ = 1 and 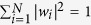. Due to only *M* (1 ≤ *M* ≤ *N*) qubits interacting with their own amplitude-damping channels respectively, then the initial state 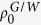 evolves in time into a mixed state 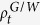 acquired simply by the composition of *M* individual maps


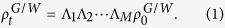


In the next section, we mainly study the QSLT of the N-qubit entangled state. So we need to start with the definition of the QSLT for an open quantum system. The QSLT can effectually define the bound of minimal evolution time for arbitrary initial states, and be helpful to analyze the maximal evolution speed of an open quantum system. A unified lower bound, including both MT and ML types, has been derived by Deffner and Lutz^26^. This QSLT is determined by an initial state *ρ*_0_ = |*ϕ*_0_〉〈*ϕ*_0_| and its target state *ρ*_*τ*_. With the help of the von Neumann trace inequality and the Cauchy-Schwarz inequality, the QSLT is as follows,


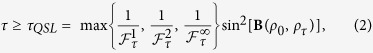


with 
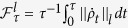
, and 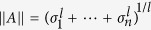 denotes the Schatten *l*-norm, *σ*_1_, *σ*_2_, ···, *σ*_*n*_ are the singular values of *A*, 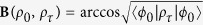 denotes the Bures angle between the initial and target states of the quantum system. And the ML-type bound based on the operator norm (*l* = ∞, that is 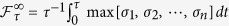) of the nonunitary generator provides the sharpest bound on the QSLT^26^. So in the following we use this ML-type bound to demonstrate the QSLT of the dynamics evolution from an initial state 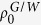 to a final state 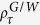 by fixing an actual evolution time *τ*. According to ref. 31, *τ*_*QSL*_/*τ* = 1 indicates the evolution is already along the fastest path and possesses no potential capacity for further quantum speedup. While for the case *τ*_*QSL*_/*τ* < 1, the speedup evolution may occur, and the much shorter *τ*_*QSL*_ the greater the capacity for potential speedup will be.

### QSLTs of N-qubit GHZ-type states

We choose GHZ-type state 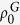 to be the initial qubits’ state. Using Eq. (1), we can straightforwardly reach the evolutional density matrix as follows


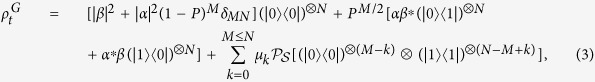


with *δ*_*MN*_ = 1 if *M* = *N*, and *δ*_*MN*_ = 0 when *M* < *N*. Owing to only *M* qubits coupled to their own noise channels, we can clearly obtain that, the off-diagonal elements of 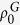 should be multiplied by the factor *P*^*M*/2^. And the diagonal terms (|0〉〈0|)^⊗*N*^ and (|1〉〈1|)^⊗*N*^ in turn give rise to new diagonal terms of the form (|0〉〈0|)^⊗(*M*−*k*)^ ⊗ (|1〉〈1|)^⊗(*N*−*M*+*k*)^, for 0 ≤ *k* ≤ *M*, and 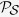 accounting for all possible permutations of the state of *M* qubits, and the coefficients *μ*_*k*_ = |*α*|^2^*P*^*k*^(1 − *P*)^*M*−*k*^.

**Figure 2:**
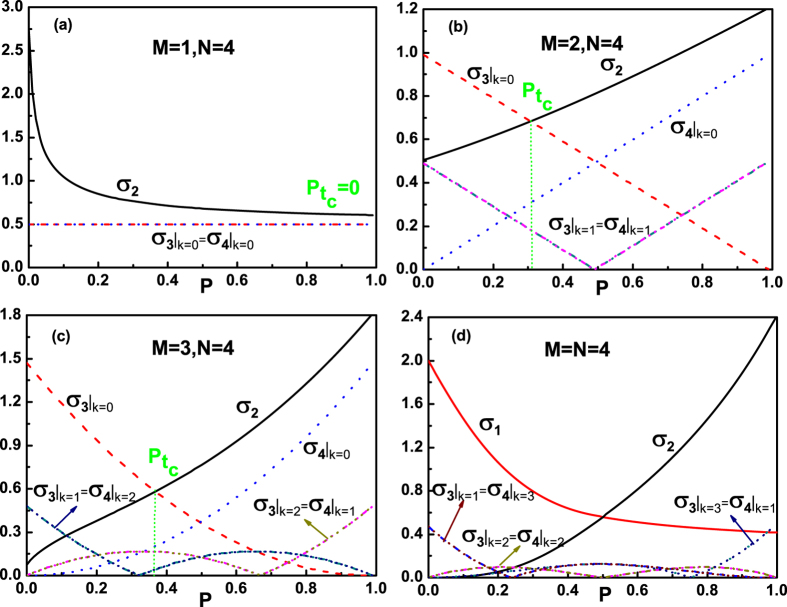
The singular value 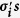 for 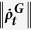 as a function of *P*_*t*_ with the different number of noise channels *M* for 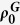 = 1/2(|1111〉 + |0000〉)(〈1111| + 〈0000|). Since all 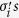 contain 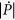, the behaviors of 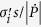 can explore the largest singular value *σ*_max_. (**a–c**) for the case *M* < *N*, there exists a certain critical strength of 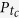; (**d**) for the case *M* = *N*, a unified expression for 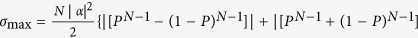

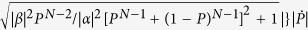
 can be acquired by combining *σ*_1_ and *σ*_2_.

In order to illustrate the roles of the number of qubits *N*, the number of noise channels *M* and the entanglement of the initial state on the quantum evolution speed of the multiqubit open system, we should firstly use the ML-type bound to calculate QSLT of the dynamics evolution from an initial state 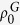 to a final state 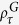 by an actual evolution time *τ*. According to Eq. (3), we can clearly find, 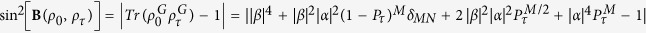. Thus our main task in the following is to calculate the singular values of 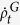 and find out the largest singular value 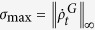. (*i*) If one consider *N* qubits interacting with independent noise channels (*M* = *N*), the singular values *σ*_*i*_ are 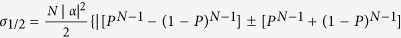

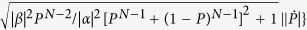
, 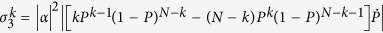, and 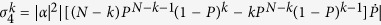, here *k* = 1, ···, *N* − 1. In the whole dynamics process, with the analysis of 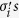 as shown in Fig. 2(d), the largest singular value *σ*_*max*_ can be given by 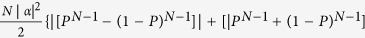

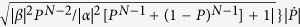
 with 0 < *P* < 1. (*ii*) For the case *M* < *N*, the singular values *σ*_*i*_ are 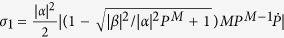, 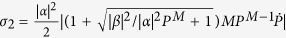, 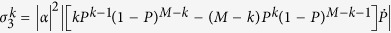, and 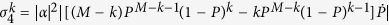, here *k* = 0, 1, ···, *M* − 1. Through comparing the above singular values, the largest singular value *σ*_max_ can be given by


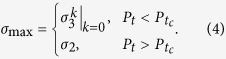


From Eq. (4), it is worth noting that, the largest singular value *σ*_max_ can occur a sudden transition from one to another at a certain critical strength of 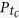 for an arbitrary *N* and *M*. And 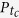 is obtained by 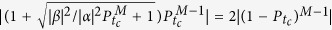. So 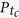 is related to the initial state (*α*, *β*) and the number of noise channels *M*. When 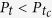, *σ*_max_ is equal to 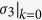, while for 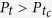, *σ*_2_ is the largest singular value of 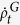 among all 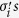. This remarkable behavior can be shown in Fig. 2 by taking the four-qubit system as an example. Therefore, when the number of noise channels *M* is less than the number of qubits *N*, the QSLT can be calculated as


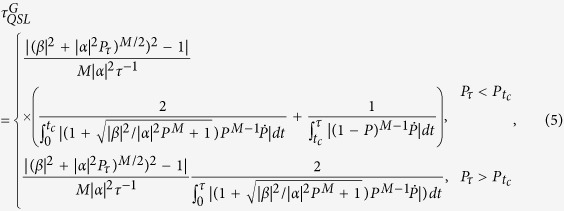


with *P*_*τ*_ means the excited population of the final state 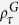. It is clear to find that the QSLT of the multiqubit GHZ-type states is evaluated as a function of the number of noise channels *M* and the initial entanglement (*α*, *β*).

**Figure 3:**
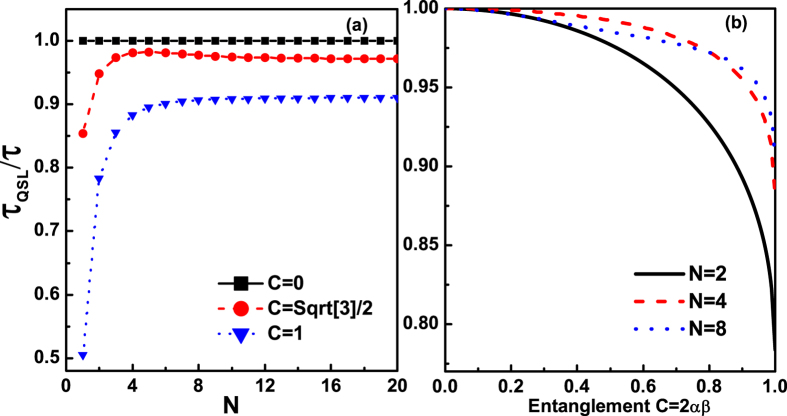
The QSLT for the evolution from 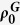 to 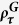 by a fixed actual evolution time *P*_*τ*_ = 0.5, quantified b *τ*_*QSL*_/*τ* as a function of the parameters for the number of qubits *N* and the entanglement *C* = 2|*αβ*| of the initially prepared state 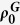, in the case *M* = *N*. Different initial states, (*α* = 1, *β* = 0), (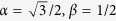) and (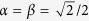) considered in (**a**); and different number of qubits, *N* = 2, 4, 8 chosen in (**b**).

**Figure 4:**
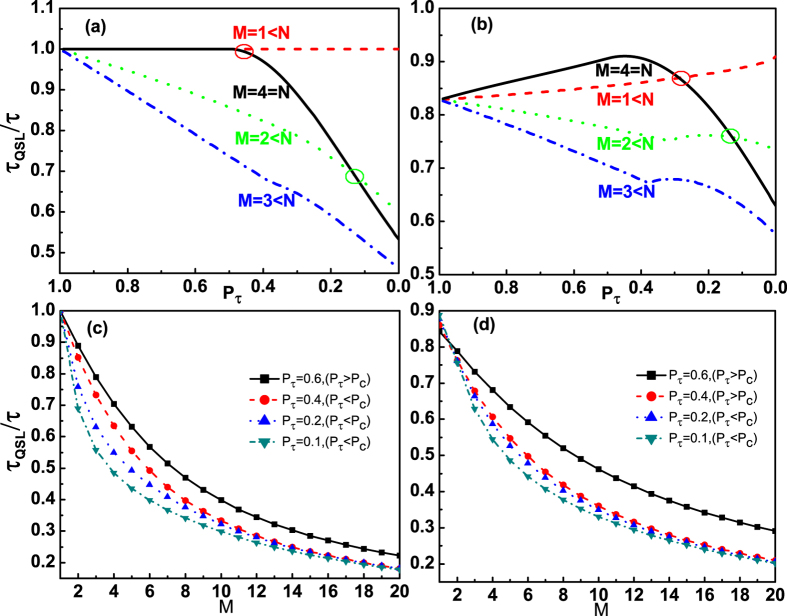
The QSLT for a given GHZ-type multiqubit state 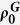, quantified by *τ*_*QSL*_/*τ* as a function of the excited population *P*_*τ*_ of the final state and the number of noise channels *M*, in the case *M* < *N*. (**a,c**) for the initial unentangled state, *α* = 1, *β* = 0; (**b,d**) for the initial entangled state 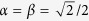.

By fixing an actual evolution time *τ*, the influences of the number of qubits *N*, the number of noise channels *M* and the entanglement of the initial state on the QSLTs for the multiqubit GHZ-type states are depicted in Fig. 3 for *M* = *N* and Fig. 4 for *M* < *N*. The entanglement of the mulitqubit state can be characterized by the genuinely multiqubit (GM) concurrence *C* defined in^41^,^42^, with *C* = 0 for a separable state and *C* = 1 for a maximally entangled state. For the biqubit system, the GM concurrence can be simplified to the Wootter’s concurrence^42^. For the N-qubit state 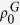, the GM concurrence can be immediately obtained *C* = 2|*αβ*|. By considering *N* qubits coupled to their independent noise channels, respectively, Fig. 3(a) clearly shows that the QSLT equals to the actual evolution time *τ* for the separable state (*α* = 1, *β* = 0). So the evolution speed of the unentangled N-qubit state 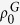 under the amplitude-damping channels is unaffected by the number of qubits *N*. While for the entangled N-qubit state 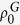, the QSLT firstly increases as the number of qubits *N* increasing and then maintains to a fixed value. That is to say, for the GHZ-type state with a given entanglement, the increasing qubits’ number *N* of the multiqubit system can lead to the smaller quantum speed in the small-scale system. However, for the larger-scale system, the evolution speed of the entangled GHZ-type state is independent of *N*. Besides, another meaningful result can be acquired from Fig. 3(b): for the entanglement GHZ-type state, the larger initial entanglement can lead to the greater potential speedup of the evolution process, and thus reduce the QSLT below its value of the unentangled multiqubit system.

Furthermore, for a given GHZ-type multiqubit state 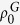 (fixing *N*, *α* and *β*), when we consider only *M* qubits coupling to their own noise channels, here *M* < *N*, the QSLT of the dynamics evolution from 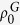 to 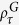 can be calculated by Eq. (5). From Fig. 4(a) for the initial unentangled state (*N* = 4, *α* = 1 and *β* = 0) and Fig. 4(b) for the initial entangled state (*N* = 4 and 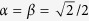), it is worth noting that the quantum speedup evolution from 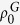 to 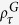 can occur at a certain region 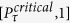 in the case *M* < *N* than the case *M* = *N*. But when only one qubit is interacting with its noise channel, the evolution speed is not accelerated for the initial unentangled state, as shown by the red dashed line in Fig. 4(a). So we therefore reach the interesting result that the speedup of the evolution of the multiqubit GHZ-type state can be acquired by controlling the number of the applied noise channels *M* < *N*. And then, numerical calculation also shows that the critical excited population 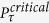 of the final state 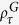 is determined by *M*. Taking the cases in Fig. 4(b) for the initial entangled state 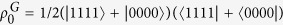 as examples, when *M* = 1, we find the value of the critical excited population is 
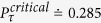
; While for *M* = 2 and *M* = 3, we can acquire 
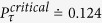
 and 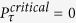.

Due to the above results in the case *M* < *N*, we should further understand the role of the number of noise channels *M* on the QSLT for a given initial state by a fixed actual evolution time *τ*. Figure 4(c,d) present the results of our analysis for *τ*_*QSL*_/*τ* as a function of the number of noise channels *M* by choosing different actual evolution times *τ*, in the case *M* < *N* = 21. By gradually increasing the number of the applied noise channels to the multiqubit system, we observe that the QSLT for the open system can monotonically decrease. That is to say, for the case of *M* < *N*, the capacity for potential speedup of evolution from 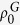 to 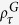 can be enhanced as the number of the applied noise channels increasing. Then the greatest capacity for quantum speedup of a given N-qubit GHZ-type state can be acquired by choosing the case *N* − 1 qubits respectively interacting with their own noise channels.

### QSLTs of N-qubit W-type states

In the following, instead of the initial GHZ-type states, we choose the W-type states as the initial N-qubit states. Only *M* qubits among the multiqubit system is independently coupled with an amplitude-damping channel, i.e., the number of noise channels *M* is less than the number of the qubits *N*. According to Eq. (1), the evolutional density matrix of the N-qubit system can be obtained


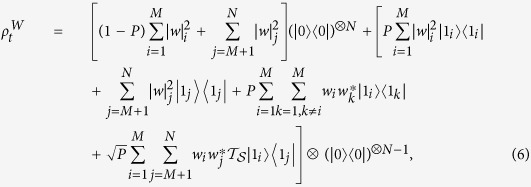


here 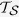 accounting for the permutation between |1_*i*_〉 and |1_*j*_〉. Next, by calculating the QSLT in Eq. (2) for the evolution from 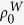 to a final state 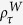 with an actual evolution time *τ*, we illustrate the influences of various parameters (*N*, *M*, and the initial entanglement parameters 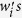) on the QSLTs of the W-type states.

In the case *M* = *N* (All qubits independently coupled to their own noise channels), the evolutional density matrix of the N-qubit system can be rewritten as 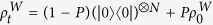, the largest singular value of 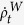 is 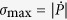, and the distance satisfies 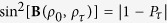. Then we acquire 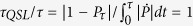. It is easy to check that if the multiqubit open system is initially prepared in the W-type state, the QSLT for the evolution from 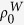 to 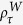 is independent of the number of qubits *N* and the initial entanglement parameters 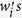. This can be understood that, when all qubits coupled to their independent noise channels, the quantum evolution speeds of the W-type multiqubit entanglement states would not be accelerated, and unaffected by the qubits’ number and the initial entanglement.

**Figure 5:**
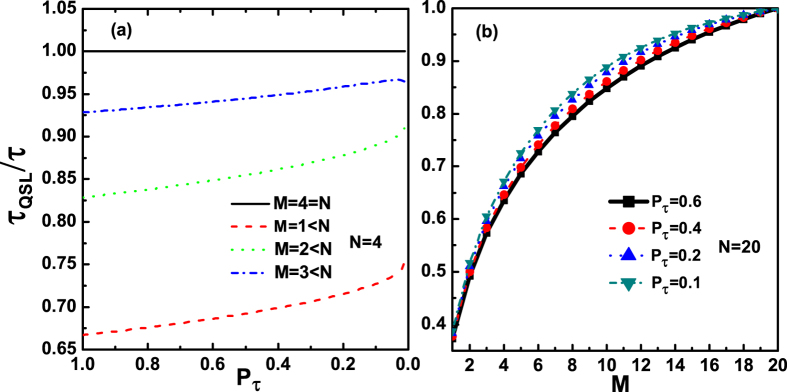
The QSLT for a given W-type multiqubit state 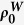, quantified by *τ*_*QSL*_/*τ* as a function of the excited population *P*_*τ*_ of the final state and the number of noise channels *M*. Here, parameters are chosen as 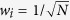, *i* = 1, 2, …, *N*.

However, for the case only *M* qubits coupled with their own noise channels, respectively, we mainly study the relationship between the number of noise channels *M* and the QSLT for a given initial W-type state 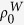, here 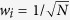, *i* = 1, 2, …, *N*. In this case, we can calculate the largest singular value 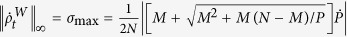 and the distance 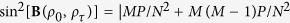

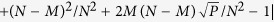
. According to the definition in Eq. (2), we can obviously find that the QSLT for a given initial W-type state is closely related to the number of noise channels *M*. Figure 5 shows the QSLT for the evolution process within a fixed actual evolution time *τ* as a function of the excited population *P*_*τ*_ of the final state and the number of noise channels *M*. By considering a given initial W-type state, we observe that, when the number of the applied noise channels is less than the number of the multiqubit system (*M* < *N*), the QSLT can be reduced, as shown in Fig. 5(a). On the other hand, a monotonic behavior of the QSLT can also be depicted in Fig. 5(b): when *M* < *N*, the QSLT for the open system can monotonically increase by gradually increasing the number of the applied noise channels to the *N*-qubit system. So we can conclude that the capacity for potential speedup of evolution from 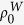 to 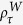 can be promoted by decreasing the number of the applied noise channels. And when only one qubit among the N-qubit system (*M* = 1) is coupled with its own noise channel, the maximal capacity for potential speedup of a given N-qubit W-type state would be reached. Finally, by comparing the analysis of the QSLT for the GHZ-type state and the W-type state, the role of the number of the applied noise channels *M* on the quantum speedup for the above two states in the case *M* < *N*, is clearly contrary, as shown in Figs 4(d) and 5(b).

## Discussion

Above all, the exemplary states we take to analyze the quantum evolution speed of multiqubit open system are the GHZ-type state and W-type state. Although these two types states represent just the restricted class of states, the study of their quantum evolution speed is important in their own right: they are crucial in quantum information and communication theory^35^,^36^,^43^,^44^,^45^,^46^, and such states have been experimentally produced in atomic and photonic systems^46^,^47^. And these two types of multiqubit states and the amplitude damping channels can be realized by the potential candidates such as cavity QED^48^, trapped ions^49^, superconducting qubits^50^ and the Nitrogen-Vacancy center of diamond^51^.

In summary, we have demonstrated the QSLT of the N-qubit entanglement state (GHZ-type state or W-type state) under amplitude-damping channels. Although a similar study of QSLT for open multiqubit system has been analyzed in the case each qubit respectively interacting with its own noise channel (*M* = *N*), the investigations mainly focus on the QSLT of a few special states (such as two-qubit Bell states, the multiqubit product state 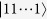), and do not concern the role of the number of the qubits N on the QSLT^31^. Here, by considering the controllable noise channels number *M*, we have clearly illustrated the roles of the number of qubits *N*, the number of noise channels M and the entanglement of the initial state on the QSLT of the multiqubit open system. The model with controllable noisy channel number plays an important role in the study of quantum metrology^52^. Some new and interesting phenomena are observed. For the case *M* = *N*, we have obtained that the QSLT of the entangled GHZ-type state first increases as the number of quits N increasing and then saturates at a fixed value. And the entanglement of GHZ-type state can shorten the QSLT of the evolution process. But the QSLT of the W-type state is independent of the number of qubits *N* and the initial entanglement. Moreover, for the other case *M* < *N*, the QSLTs of the mulitqubit GHZ-type states and W-type states are shorter than in the case *N* qubits independently coupled with independent noise channels. So the speedup of a dynamics process of a given N-qubit GHZ-type state or W-type state occurs when the controllable noise channels’ number is less than the number of qubits. Our results may be of both theoretical and experimental interests in exploring the potential quantum speedup for the multiqubit states by the controllable noise channels’ number in the large-scale information processing.

## Additional Information

**How to cite this article**: Zhang, Y.-J. *et al*. Speedup of quantum evolution of multiqubit entanglement states. *Sci. Rep.* **6**, 27349; doi: 10.1038/srep27349 (2016).

## Figures and Tables

**Figure 1:**
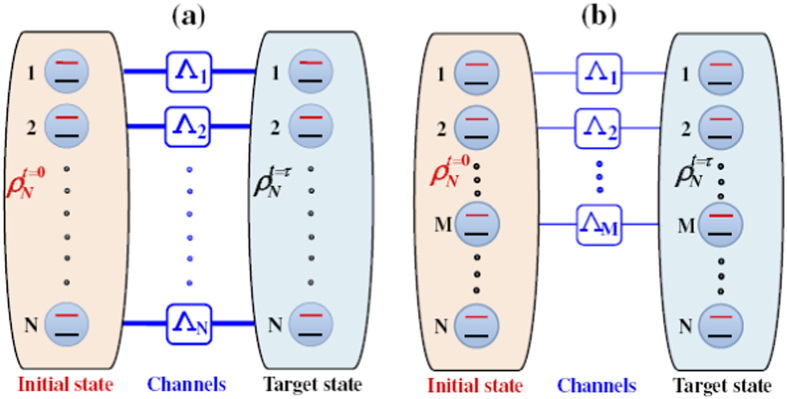
Quantum evolution speed for multiqubit entanglement states in two different cases. The map Λ means a completely positive trace-preserving noise channel. (**a**) one case is described that *N* qubits would be coupled with independent noise channels Λ′*s*; (**b**) the other case of only *M* qubits among the N-qubit system respectively interacting with their own noise channel Λ, here the number of the noise channels *M* is less than the number of the qubits *N*.
